# Deliberate reasoning is not affected by language

**DOI:** 10.1371/journal.pone.0211428

**Published:** 2019-01-31

**Authors:** Martin Jensen Mækelæ, Gerit Pfuhl

**Affiliations:** Department of Psychology, Faculty of Health Sciences, UiT The Arctic University of Norway, Tromsø, Norway; Middlesex University, UNITED KINGDOM

## Abstract

**Background:**

Millions of people use a second language every day. Does this have an effect on their decision-making? Are decisions in a second language more deliberate? Two mechanisms have been proposed: reduced emotionality or increased deliberation. Most studies so far used problems where both mechanisms could contribute to a foreign language effect. Here, we aimed to identify whether deliberate reasoning increases for problems that are devoid of any emotional connotation when using a second language or having to switch between native and second language.

**Method:**

We measured deliberate reasoning with items from the cognitive reflection test, ratio bias, a probability matching task, and base rate neglect items. We recruited over 500 participants from Norway and the Netherlands that had English as their second language. Participants were randomly assigned to either the native, switching or second language condition. We measured: number of correctly answered items–deliberate reasoning score, perceived effort, perceived accuracy or confidence, and language proficiency.

**Results:**

Deliberate reasoning was not increased when using a second language or when having to switch between native and second language. All three groups performed equally well. Significant predictors of deliberate reasoning were age, gender, education, perceived effort, and confidence but not the language context. Participants with low English proficiency spent more time reading compared to more fluent speakers.

**Conclusion:**

There is no advantage of second language on deliberate reasoning in the absence of time pressure. Deliberation was not increased by providing items in a second language, but through the willingness to spend cognitive effort and time to read carefully.

## Introduction

Millions of people make decisions in a second language, and global trade and international agreements rely on second language proficiency. These decisions should be made carefully and wisely. However, human decision-making is prone to bias and systematic errors [1]. Is the use of a second language an advantage or a disadvantage for making rational decisions? That is, are we reasoning more deliberately when thinking in a second language?

Since, “human rational behavior is shaped by a scissors whose two blades are the structure of task environments and the computational capabilities of the actor” [2] we here investigated whether the language environment influences our decision-making abilities. Recent studies found that reasoning is more rational in a foreign language context ([3, 4]). This has been explained with reduced emotionality or increased deliberation when problems are presented in a foreign language ([3, 5]). Both, reduced emotionality and increased deliberation descend from the more general dual process theories of human thinking.

### Dual-process theories and decision-making

Dual-processing theories divide human thinking into two separate systems [6–8]. System 1 or intuitive thinking is autonomous, automatic, fast, parallel, unconscious and effortless. System 2 or deliberate reasoning is slow, serial conscious and require effort. Since deliberate reasoning is costly, relying on intuitive processes to save mental effort is a universal phenomenon in humans [9]. Accordingly, there is a trade-off between intuitive and deliberate reasoning. Relying on intuition is in many cases sufficient and adaptive [10, 11], but can also lead to irrational decisions [12]. According to Stanovich [13], the capacity to execute deliberate reasoning is separate from the ability to know when and how to apply deliberate reasoning. Individual differences in rationality can occur due to differences in intelligence or thinking disposition. Thinking disposition refers to a person’s cognitive style as in favoring either using system 1 or system 2. This can be assessed through self-report questionnaires like the need for cognition scale [14] or it can be tested in reasoning tasks such as the cognitive reflection test (CRT) ([15]). The defining feature in these reasoning tasks is that the items elicit an automatic incorrect response, and one has to first detect the conflict and then inhibit the intuitive wrong response and answer with a second deliberate response [16]. Scoring low on the CRT corresponds to an intuitive cognitive style, whereas scoring high on the CRT corresponds to a deliberative cognitive style [17].

Deliberate reasoning and rational decision-making depend on a range of factors, i.e. differences in thinking disposition, age, socio-economic status, as well as our current mood [18–20]. Accordingly, knowing which factors enhance deliberation and override intuitive responses when detecting a conflict would be beneficial, and these factors could potentially serve as triggers. One such promising “enhancer” might be language. Indeed, there are reports of bilingualism improving executive functions [21], though this has recently been questioned [22]. Still, the inhibitory control processes required to speak two languages might generalize to non-linguistic domains.

### The influence of the language environment on deliberate reasoning

In bilinguals the language environment, using either their native language or a foreign language, may influence decision-making and emotional reactions. In a classical decision-making paradigm, Keysar et al. [4] found a reduced “framing effect” in the second language condition, i.e. participants’ choices were more similar for whether a situation was described either as loss or gain. Subsequently, a series of studies have investigated how using a second language influences our decisions. Considering moral dilemmas in a foreign language resulted in more utilitarian choices [3], and more lenient judgement towards moral transgressions [23, 24]. When assessing the risk of potential hazards of activities like “traveling by airplane” and “biotechnology” the risks associated with them were rated as lower and the benefits as larger when presented in a second language [25]. People using a second language also show more consistent risk preferences, which is reflected in both the Asian Disease paradigm [4] and the Holt–Laury test [3, 5]. Using a second language also reduced the “hot hand fallacy” [26]. For these findings two possible explanations have been proposed.

The first explanation is reduced emotionality when using a non-native language. Bilinguals have shown to be less emotionally reactive when using a foreign language than when using their native language [27, 28]. In decision-making emotions often play an informative role [20], and trigger the more intuitive system 1 [29]. Such emotions can in some situations lead to biased decision-making and impede rationality [30]. Accordingly, when emotionality is reduced, biases associated with emotions will be diminished. Emotional distance might reduce automatic processing which again prompts and allows people to rely more on deliberative processing ([3, 4]).

The second explanation is reduced automaticity or increased deliberation when using a non-native language. This account proposes that reduced cognitive fluency might activate deliberate reasoning, i.e. thinking in a second language might not be as fluent as native thinking and the cognitive dysfluency might prompt people to process the information more carefully, deeply, and abstractly. Or, thinking in a second language might require more effort and as a side-effect System 2 is more engaged than when the same task is presented in one’s native language. Note, we cannot yet separate whether the effects are caused by increased deliberation or reduced intuition [24] or both. Supporting the increased deliberation account are previous studies showing that metacognitive experiences of difficulty or disfluency appear to serve as an alarm that activates analytic forms of reasoning [31].

If effort triggers more deliberate thinking then participants highly fluent in a second language may not show a foreign language effect. Indeed, that is what Oganian, Korn, and Heekeren [32] found. A reduction of the “framing effect” in the foreign language only appeared in the language switching condition but not in the second language group. The authors attributed this to enhanced cognitive control. An even better test of the increased deliberation account would be a task without any emotional connotation. Costa et al. [5] used the Cognitive reflection test, and found no difference when testing over 640 participants, either native Spanish or native English, in their native or second language. However, the performance was generally low and their participants were quite fluent in their second language. Recently, [33] tested three well-known reasoning biases. Neither the outcome bias, the conjunction fallacy nor the base-rate neglect fallacies were reduced in the second language condition. However, it is still possible that logical reasoning can be enhanced by presenting items in a language switching context. Even for persons with high language proficiency switching is more demanding than non-switching.

### Assessing the effect of language on deliberate reasoning

To test the increased deliberation/reduced intuition account and the language-switching hypothesis, we measured if deliberative reasoning increases in a non-native language context. We used a battery of deliberative reasoning tasks, all devoid of emotional content. We had three conditions, native language, second (foreign) language, and a switching language condition (alternating between native and second language). Second language is here defined as a language learned after early childhood (age 1–3 years) following the acquisition of native language (and will replace the use of foreign language from this point). We used the NASA task load index to assess if more effort was required in the second language or the switching language condition. We also measured self-rated English proficiency and overconfidence.

#### We had two primary hypotheses

1) participants in the second language condition will score higher on the deliberative reasoning composite than participants in the native language condition. 2) Participants in the switching language condition will score higher on the deliberative reasoning composite than participants in the native language condition.

#### Secondary hypotheses

3) Participants in the second language condition will report higher effort expenditure than participants in the native language condition. 4) The participants in the switching language condition will report higher effort expenditure than both single language conditions. 5) Overconfidence is higher in the native language condition than the other two conditions.

#### Exploratory hypotheses

6) The switching language condition will differ from the second language condition on deliberate reasoning composite, non-directional hypothesis. 7) The switching language condition will differ from the second language condition on effort expenditure, non-directional hypothesis. 8) Effort expenditure correlates with deliberative reasoning composite, non-directional hypothesis. 9) There is an interaction between language condition and effort, which will influence deliberative reasoning composite, non-directional. 10) Second language proficiency correlates with deliberative reasoning composite, non-directional.

Hypotheses were classified into primary, secondary and explorative hypotheses to comply with new publishing guidelines [34]. The content and directions of our hypotheses remain the same.

## Methods

### Preregistration

This study was pre-registered on the Open Science Framework (OSF) (https://osf.io/qystj/). Additional details regarding the timing of the behavioral protocols and all raw data can be found on OSF.

### Participants and procedure

526 participants completed the survey. 53 participants were excluded due to language reasons (exclusion criteria pre-registered). Of the 53 excluded participants; 22 participants reported Dutch/Norwegian was not their native language, 20 participants reported more than one native language, 11 reported self-rated English proficiency < 2 (range 1–7). Results did not change with the 53 participants included. Demographic information for the 473 remaining participants is presented in Table 1 in the results section.

**Table 1:** summary statistics mean (SD) for the 3 conditions.

	Native language	Switching languages	English (second) language
Education (1–4)	1.87 (.9)	2.05 (.92)	1.93 (.85)
Age in years	32.29 (11.15)	31.76 (11.53)	30.86 (11.66)
Gender male / female	85 / 76	78 / 76	80 / 78
Language proficiency (1–7)	5.53 (1.08)	5.39 (1.06)	5.46 (1.07)
Deliberate reasoning score (0–16)	11.14 (3.92)	11.01 (3.65)	11.22 (3.37)
Modified CRT score (0–7)	4.16 (1.87)	4.18 (1.8)	4.19 (1.85)
Perceived effort (0–100)	50.82 (14.51)	48.94 (18.05)	52.15 (14.51)
Overconfidence in %	.97 (.58)	.93 (.32)	.90 (.41)

### Materials

#### Deliberate reasoning

We used four different reasoning tasks from the heuristics and bias literature. All tasks are designed to create a conflict between a heuristic, wrong answer, and a correct answer that can be reached through deliberate reasoning.

#### The Cognitive reflection test

The items are designed to prompt an intuitive wrong answer, which must be overridden by a deliberate correct answer. We used items 2–6 from [35] and replaced the first and last item with the first two items from Thomson and Oppenheimer [36], resulting in a modified CRT of 7 items. We replaced the first and last item from Toplak et al. as pilot data yielded that these items are too well known or were affected by age and economic knowledge.

#### Base-rate neglect

Items are designed to create a conflict between a character description, which fits a stereotype (prompting a heuristic answer), and the base-rate information provided in the question. In order to answer the question correctly, one must use the base-rate information and not rely solely on the character description. We used six conflict items and two neutral items [37, 38]. The neutral items served to disguise the nature of the task and were scored as correct irrespective of the participants’ choice. The results do not differ if these neutral items are scored 0, as it was preregistered. We deviate from our pre-registration as participants were asked to make the judgement of how many items they got correct (confidence) based on the total number of items.

#### Ratio bias

We used two items from [39]. In this task, participants are presented with two jars filled with red and white beads. They win if they can draw a red bead. They are asked to choose from which jar they would like to draw a bead (without looking into the jars). The proportion of beads is manipulated such that there are more red beads in one jar (heuristic, wrong answer), but a higher proportion of the beads are red in the other jar (deliberative, correct answer).

#### Probability matching task

In this task subjects are presented with ten pairs of cups, each pair consists of two colors (blue and yellow, here). They are told that 10 five-dollar bills are hidden under 10 of the 20 cups. 7 of the five-dollar bills are hidden under blue cups and 3 are hidden under yellow cups. Participants are then asked to choose one cup from each pair. Most people choose a matching strategy, choosing 7 blue cups and three yellow cups (heuristic, wrong answer). However, a maximizing strategy, choosing 10 blue cups (deliberate, correct answer), provides better odds [40].

The deliberative reasoning score ranged from 0–16 with each deliberate reasoning problem scored as correct = 1 or incorrect = 0.

#### Effort

Effort was measured with the NASA task load index [41]. Effort is self-assessed and scored along six dimensions: mental, physical, temporal, performance, effort, frustration. Participants rated their effort on a 0–100 scale in steps of 5 (range 0–21) where 0 was “very low” and 21 was “very high”. Perceived effort was calculated by using the average score of the mental effort and performance effort scales from the NASA task load index.

#### Language proficiency

Language proficiency was measured on a 7-point Likert scale from “understand a few words” to “Master it like native language”.

#### Overconfidence

Participants gave a subjective rating of how many correct answers they had on a scale from 0–16. The overconfidence score was calculated as the discrepancy between how many items participants think they got correct versus their actual deliberate reasoning score, expressed as % (e.g. think got 15 items correct, did get 10 items correct, then it is 15/10 or 150% overconfident).

### Procedure

The survey was created in Qualtrics (Qualtrics, Provo, UT). The survey was spread through social media and snowballing in Norway and the Netherlands. There were four versions of the survey in both the Norwegian and the Dutch sample. One survey was in Norwegian or Dutch (native language), respectively; one survey was in English (second language), and a third survey had alternate items in native and second language (switching language), the fourth survey was the same as the third (switching) but with opposite items in native and second language. Participants were automatically randomized among the four conditions, with equal numbers of participants in the native, second, and the switching condition. The survey was broadcast as a test of problem-solving skills and participants received their score as feedback. The test started with a consent form and ended with a short debriefing form that included a description of system 1 and system 2, and how bias can lead to errors in decision-making. The experimental manipulation of language was not mentioned.

### Ethics

The project was evaluated by the Norwegian Center for research data (NSD) and required no notification (Ref 2017/52276 / 3 / BGH) according to the regulations. The IRB at the Department of Psychology at UiT The Arctic University of Norway approved the study.

### Sample size calculations

Our sample size is based on a small expected effect size of f = .15 (based on [3]), a type I error of 5% and a type II error of 20%. Using G power 3.1 [42] an N of at least 432 was required to find a difference between the three language conditions (one-way ANOVA). Furthermore, our directional analyses and comparing two conditions (post-hoc tests and t-tests) yielded a required sample size of 199 participants per condition, or nearly 600 participants in total.

### Planned statistical analyses

For hypothesis 1, 2 and 6: one-way ANOVA with deliberative reasoning score as outcome measure and group, native, mixed, second language as predictor, and post-hoc comparisons.

For hypothesis 3 and 4: one-way ANOVA with perceived effort as outcome measure and group, native, mixed, second language as predictor, and post-hoc comparisons.

For hypothesis 5: one-way ANOVA with overconfidence score as outcome measure and group, native, mixed, second language as predictor.

For hypothesis 7: Person correlation/simple regression between effort expenditure and deliberative reasoning score.

For hypothesis 8: ANCOVA with deliberative reasoning score as outcome measure and group native, mixed, second language as predictor and perceived effort as co-variate

For hypothesis 9: Pearson correlation/simple regression between language proficiency and deliberative reasoning score.

### Additional statistical analyses

Pearson correlation between deliberative reasoning score and 1) Age 2) Education 3) Gender. We also coded CRT answers as intuitive response, correct or other erroneous response and compared whether the groups differ in the type of error made. We, furthermore, calculated a deliberate reasoning score solely of the seven CRT items and compared the groups. These two analyses are based on Costa et al. [5]. Finally, we also looked at the latency before answering (using the time stamp ‘first click’ in Qualtrics) for all 16 items and compared those latencies by group and language proficiency.

If the assumptions for parametric tests are violated (visual inspection of residuals and test of homogeneity) we performed non-parametric tests. For the non-parametric tests we report effect sizes from the parametric tests. All tests were performed in jasp (jasp-stats.org).

### Open science

Material and data files are available at https://osf.io/5gcnh/

## Results

The groups did not differ in their age, education, gender composition or language proficiency.

Hypothesis 1, 2 and 6. We performed a Kruskal-Wallis ANOVA with deliberate reasoning score as outcome measure and group (native, switching, second language) as predictor. A one-way ANOVA yielded no statistically significant group difference χ^2^(2) = 0.43, *P* = .806, η^2^ = .001. The data did not support hypothesis 1, 2 and 6. There was no group difference in deliberate reasoning across different language contexts.

Hypothesis 3 and 4. We performed a Kruskal-Wallis ANOVA with perceived effort as outcome measure and group (native, switching, second language) as predictor. A one-way ANOVA yielded no statistically significant group difference χ^2^(2) = 1.30, *P* = .522, η^2^ = .007. This shows that hypothesis 3 and 4 were not supported. There was no difference in perceived effort across different language contexts.

Hypothesis 5. We performed a Kruskal-Wallis ANOVA with overconfidence score as outcome measure and language condition as predictor. A one-way ANOVA showed no statistically significant group differences: χ^2^(2) = 0.34, *P* = .845, η^2^ = .004. This means hypothesis 5 was not supported. There was no difference in overconfidence between the language conditions.

Hypothesis 7. A Spearman correlation between deliberative reasoning score and perceived effort resulted in a small positive significant correlation *r*_*s*_(473) = .23, *P* < .001. There is a small positive relationship between perceived effort in solving tasks and deliberate reasoning.

Hypothesis 8. An ANCOVA showed no significant effect of language condition on deliberate reasoning score after controlling for perceived effort; main effect of group: F(2,469) = 0.007, *P* = 0.993, η^2^ = 0. However, there was a large effect of perceived effort on the deliberate reasoning score, F(1, 469) = 24.043, *P* < .001, η^2^ = .049. Hypothesis 8 was not supported. Perceived effort, not language explained some variance in the deliberate reasoning score.

Hypothesis 9. A Spearman correlation between self-rated English proficiency and deliberate reasoning score showed a significant small positive correlation *r*_*s*_ (473) = .17, *P* < 001. Further analysis assessing each language condition separately revealed that only the second language condition showed a significant positive correlation between English proficiency and deliberate reasoning *r*_*s*_ (158) = .24, *P* = .002, while the native language condition *r*_*s*_ (154) = .11, *P* = .156, and the switching language condition *r*_*s*_ = .14, *P* = .078, were not significant. Those who were less fluent in their second language did not perform as well as their more fluent peers on deliberate reasoning tasks.

### Additional, explorative analysis

Following [5] we classified items from the CRT as either heuristic response, correct response, or other error. If the language context reduces intuitive or heuristic responses then there might be a group difference in the type of errors. The type of error did not differ between the groups (heuristic errors: χ^2^ = 15.2, *P* = .364, other errors: χ^2^ = 10.61, *P* = .389).

We also measured latency before making a decision, we summed up those latencies for all 16 items. We found no group difference, F(2,427) = .088, *P* = .916, η^2^ = 0, but those with self-judged lower second language proficiency (median split applied) spent more time before answering, F(1,427) = 4.132, *P* = .043, η^2^ = .01.

A Spearman correlation showed a small significant negative relationship between age and deliberate reasoning score *r*_*s*_ (473) = -.19, p < .001. Older participants scored lower on the deliberate reasoning tasks.

A Spearman correlation showed a small significant positive relationship between education and deliberate reasoning score *r*_*s*_ (473) = .22, p < .001. Those with higher education scored higher on the deliberate reasoning tasks.

A Spearman correlation showed a small negative correlation between gender and deliberate reasoning score *r*_*s*_ (473) = -.13, *P* = .005. Men scored higher than women on the deliberate reasoning items.

Finally, we performed an explorative linear regression. This regression explained 35% of the deliberate reasoning score and all predictors but group and proficiency in English (self-rated) were significant predictors (see Table 2).

**Table 2:** Coefficients of predictors for deliberate reasoning score, and 95% confidence interval.

Model	Unstan-dardized	Standard Error	Standar-dized	t	p	2.5%	97.5%
(Intercept)	10.625	1.114		9.534	< .001	8.435	12.815
Perceived effort	0.033	0.009	0.146	3.776	< .001	0.016	0.050
Overconfidence	-3.389	0.324	-0.417	-10.457	< .001	-4.026	-2.752
Language group	-0.127	0.167	-0.029	-0.761	0.447	-0.455	0.201
Age in Years	-0.045	0.014	-0.143	-3.297	0.001	-0.073	-0.018
Gender	1.118	0.278	0.153	4.022	< .001	0.572	1.664
Education	0.901	0.159	0.221	5.657	< .001	0.588	1.214
English proficiency	0.249	0.140	0.073	1.775	0.077	-0.027	0.525

## Discussion

Our hypothesis was that a foreign language context or a language switching context would increase deliberate reasoning. We found no enhanced deliberation across different language contexts. This also applied after controlling for perceived effort. This is contrary to our prediction and the reduced intuition/increased deliberation account of decision-making in a foreign language. This agrees well with the previous finding from Costa et al. [5] study 4 using the three items from the CRT and the recent study [33] using three cognitive biases. Since Costa and colleagues compared native with second language it was still possible that language switching could enhance deliberation. However, our study found no enhanced logical reasoning in the switching condition either. Poor performance cannot explain the absence of the effect. Our participants had on average 11 out of the 16 items correct, and even when including solely the CRT items (N = 7) the average score was over 50% correct (see Table 1), whereas Costa et al. (2014a) found that only 17–34% had 2 or 3 items correct. Our data also questions whether switching is more demanding. We did not find higher perceived cognitive effort in the switching group than the native or second language group. This was contrary to our expectation but might be explained by our participants’ high second language proficiency [43]. There was a small positive correlation between perceived effort and deliberate reasoning.

It is possible that our framing of the task as a “test of everyday problem solving” activated analytical thinking, and subsequently, participants in all language conditions were highly motivated to answer correctly. Alternatively, not language per se but fluency may cause the second language effect. Too low fluency would be detrimental (and is an exclusion criterion), but too high fluency negates the increased deliberation account. Spending more effort could compensate a lower fluency, as our latency to make a decision data suggests. Furthermore, Meyer et al. [44] did not find any difference in analytical thinking due to disfluency as [31] did, making the increased deliberation account less likely. Indeed, the bilingual executive advantage seems not to generalize beyond the language domain [45], because “bilingual advantages in executive function depend on characteristics of the participants and features of the tasks” [21].

Overconfidence did not vary by the language context. This is incongruent with a recent study [46] who found higher overconfidence in financial literacy by individuals with English as a second language in Australia. However, their sample of non-native English speakers was small (*N* = 33) and these participants may vary on other aspects from native English speakers in Australia. Our results suggest that there is also no difference in overconfidence due to language context when there is no performance difference.

There was a small positive correlation between second language proficiency and deliberate reasoning score, reaching significance only in the foreign language condition and being only marginally significant in the switching language condition. The more fluent the better the deliberate reasoning. Higher verbal intelligence could account for the results in both conditions.

Language proficiency was high in [5] too. The authors found no foreign or second language effect in two out of the seven problems that had an emotional component. That illustrates that the effect might be rather specific to the framing/loss aversion problem (Asian disease) case. In the framing problem there is no intuitive wrong answer. This differs from the cognitive reflection task items that require both conflict detection, inhibition of the wrong response and computation of the correct response.

### Additional analyses

Our additional analysis showed that age was negatively correlated with deliberate reasoning score, meaning older participants performed slightly worse than younger participants. This could be explained in part by decreased fluid intelligence, decreased processing speed and frontal decline in older adults [47–50], or increased effort cost [51]. Future studies should investigate deliberate reasoning in older adults while controlling for these factors.

The additional analysis also showed a small positive relationship between higher education and higher deliberate reasoning scores. It remains to be seen whether those with higher education have better analytical thinking or know better when to use it [52]. We also found a small sex difference in deliberate reasoning performance where men performed slightly better than women. This has been observed in other studies with the CRT [15, 36, 53]. This has often been attributed to sex differences in numeracy where men tend to perform slightly better [15, 54] and even biological explanations have been proposed [55]. Interestingly, [53] found no sex difference on the CRT after controlling for quantitative self-efficacy.

## Limitations

There is a range of factors why deliberation was not affected by the language environment. Firstly, we tested persons that are comparatively well educated and that are proficient in English. Both in Norway and in the Netherlands, English is taught early in school and very prominent in daily life, e.g. movies are not dubbed. Secondly, using snowballing as the recruiting method could have biased us towards curious, open-minded participants, which are often also critical thinkers. Thirdly, a major difference to previous studies is our non-emotional material; and we also did not deceive participants but informed them that this is a study on problem-solving. This information could be sufficient to trigger attention and control mechanisms, needed for not answering intuitively. Indeed, participants less fluent in English did spend more time on a page before answering, indicating a motivation to do well in our tasks. As such, not the language context but knowing that we measure “thinking” may encourage participants to reason deliberately. Our results may differ if we would have applied time pressure. Intuitive responses are more prevalent under time pressure [10] and can lead to more honest behavior [56, 57].

## Conclusion

Deliberate reasoning in a second language does not make us wiser. We found similar deliberate reasoning in one’s native and a second language, and the reasoning was perceived as similarly effortful. We know that willingness to spend cognitive effort depends on many factors [31, 43, 51, 58, 59] but the language context does neither increase nor hamper deliberation. This is reassuring for trade, because social efficiency is related to deliberation [17]. Still, our data cannot exclude the possibility that a small effect of language on deliberate reasoning exists. Furthermore, decision-making in a foreign language can still be beneficial for tasks with high emotional connotation [20, 27–30] and in moral judgments [3, 23, 24, 60, 61].

## References

[1] West RF, Toplak ME, Stanovich KE. Heuristics and Biases as Measures of Critical Thinking: Associations with Cognitive Ability and Thinking Dispositions. Journal of Educational Psychology. 2008;100(4):930–41. 10.1037/a0012842

[2] Simon HA. Invariants of Human Behavior. Annual Review of Psychology. 1990;41(1):1–20. 10.1146/annurev.ps.41.020190.000245 18331187

[3] Costa A, Foucart A, Hayakawa S, Aparici M, Apesteguia J, Heafner J, et al Your Morals Depend on Language. PLOS ONE. 2014;9(4):e94842 10.1371/journal.pone.0094842 24760073 PMC3997430

[4] Keysar B, Hayakawa SL, An SG. The foreign-language effect: thinking in a foreign tongue reduces decision biases. Psychological science. 2012;23(6):661–8. Epub 2012/04/21. 10.1177/0956797611432178 . 22517192

[5] Costa A, Foucart A, Arnon I, Aparici M, Apesteguia J. “Piensa” twice: On the foreign language effect in decision making. Cognition. 2014;130(2):236–54. 10.1016/j.cognition.2013.11.010 24334107

[6] Kahneman D. Thinking, fast and slow New York, NY, US: Farrar, Straus and Giroux; 2011 499- p.

[7] Evans JSBT. Heuristic and analytic processes in reasoning*. British Journal of Psychology. 1984;75(4):451–68.

[8] Sloman SA. The empirical case for two systems of reasoning. Psychological Bulletin. 1996;119(1):3–22. 10.1037/0033-2909.119.1.3

[9] Hull CL. Principles of behavior: an introduction to behavior theory Oxford, England: Appleton-Century; 1943 x, 422–x, p.

[10] Rand DG. Cooperation, Fast and Slow: Meta-Analytic Evidence for a Theory of Social Heuristics and Self-Interested Deliberation. Psychological science. 2016;27(9):1192–206. 10.1177/0956797616654455 27422875

[11] Gigerenzer G, Gaissmaier W. Heuristic Decision Making. Annual Review of Psychology. 2011;62(1):451–82. 10.1146/annurev-psych-120709-145346 . 21126183

[12] Todd PM, Gigerenzer G. Précis of Simple heuristics that make us smart. Behavioral and Brain Sciences. 2000;23(5):727–80. 10.1017/S0140525X00003447 11301545

[13] Stanovich KE. Distinguishing the reflective, algorithmic, and autonomous minds: Is it time for a tri-process theory? In two minds: Dual processes and beyond New York, NY, US: Oxford University Press; 2009 p. 55–88.

[14] Cacioppo JT, Petty RE. The need for cognition. Journal of Personality and Social Psychology. 1982;42(1):116–31. 10.1037/0022-3514.42.1.116 7131245

[15] Frederick S. Cognitive Reflection and Decision Making. Journal of Economic Perspectives. 2005;19(4):25–42. 10.1257/089533005775196732

[16] Pennycook G, Fugelsang JA, Koehler DJ. What makes us think? A three-stage dual-process model of analytic engagement. Cognitive psychology. 2015;80:34–72. Epub 2015/06/21. 10.1016/j.cogpsych.2015.05.001 . 26091582

[17] Capraro V, Corgnet B, Espín AM, Hernán-González R. Deliberation favours social efficiency by making people disregard their relative shares: evidence from USA and India. Royal Society Open Science. 2017;4(2). 10.1098/rsos.160605 PMC5367314 28386421

[18] Bruine de Bruin W, Parker AM, Fischhoff B. Individual differences in adult decision-making competence. J Pers Soc Psychol. 2007;92(5):938–56. Epub 2007/05/09. 10.1037/0022-3514.92.5.938 . 17484614

[19] Finucane ML, Mertz CK, Slovic P, Schmidt ES. Task complexity and older adults’ decision-making competence. Psychology and aging. 2005;20(1):71–84. Epub 2005/03/17. 10.1037/0882-7974.20.1.71 . 15769215

[20] Greenspan P. Practical reasoning and emotion The Oxford handbook of rationality: Oxford, Oxford University Press; 2004 p. 206–21.

[21] Bialystok E, Poarch G, Luo L, Craik FIM. Effects of bilingualism and aging on executive function and working memory. Psychology and aging. 2014;29(3):696–705. Epub 2014/09/23. 10.1037/a0037254 . 25244487 PMC4274603

[22] Paap KR, Johnson HA, Sawi O. Bilingual advantages in executive functioning either do not exist or are restricted to very specific and undetermined circumstances. Cortex; a journal devoted to the study of the nervous system and behavior. 2015;69:265–78. Epub 2015/06/07. 10.1016/j.cortex.2015.04.014 . 26048659

[23] Geipel J, Hadjichristidis C, Surian L. Foreign language affects the contribution of intentions and outcomes to moral judgment. Cognition. 2016;154:34–9. Epub 2016/05/28. 10.1016/j.cognition.2016.05.010 . 27232522

[24] Geipel J, Hadjichristidis C, Surian L. How foreign language shapes moral judgment. Journal of Experimental Social Psychology. 2015;59:8–17. 10.1016/j.jesp.2015.02.001.

[25] Hadjichristidis C, Geipel J, Savadori L. The effect of foreign language in judgments of risk and benefit: The role of affect. Journal of experimental psychology Applied. 2015;21(2):117–29. Epub 2015/04/22. 10.1037/xap0000044 . 25893443

[26] Gao S, Zika O, Rogers RD, Thierry G. Second language feedback abolishes the "hot hand" effect during even-probability gambling. The Journal of neuroscience: the official journal of the Society for Neuroscience. 2015;35(15):5983–9. Epub 2015/04/17. 10.1523/jneurosci.3622-14.2015 . 25878271 PMC4397599

[27] AyÇIÇEgi-Dinn A, Caldwell-Harris CL. Emotion-memory effects in bilingual speakers: A levels-of-processing approach. Bilingualism: Language and Cognition. 2009;12(3):291–303. 10.1017/S1366728909990125

[28] Dewaele J-M. The Emotional Force of Swearwords and Taboo Words in the Speech of Multilinguals. Journal of Multilingual and Multicultural Development. 2004;25(2–3):204–22. 10.1080/01434630408666529

[29] Livet P. Rational choice, neuroeconomy and mixed emotions. Philosophical Transactions of the Royal Society B: Biological Sciences. 2010;365(1538):259–69. 10.1098/rstb.2009.0177 20026464 PMC2827455

[30] Lerner JS, Li Y, Valdesolo P, Kassam KS. Emotion and decision making. Annu Rev Psychol. 2015;66:799–823. Epub 2014/09/25. 10.1146/annurev-psych-010213-115043 . 25251484

[31] Alter AL, Oppenheimer DM, Epley N, Eyre RN. Overcoming intuition: metacognitive difficulty activates analytic reasoning. Journal of experimental psychology General. 2007;136(4):569–76. Epub 2007/11/15. 10.1037/0096-3445.136.4.569 . 17999571

[32] Oganian Y, Korn CW, Heekeren HR. Language switching-but not foreign language use per se-reduces the framing effect. Journal of experimental psychology Learning, memory, and cognition. 2016;42(1):140–8. Epub 2015/06/23. 10.1037/xlm0000161 . 26098182

[33] Vives M-L, Aparici M, Costa A. The limits of the foreign language effect on decision-making: The case of the outcome bias and the representativeness heuristic. PLOS ONE. 2018;13(9):e0203528 10.1371/journal.pone.0203528 30192841 PMC6128570

[34] Appelbaum M, Cooper H, Kline RB, Mayo-Wilson E, Nezu AM, Rao SM. Journal article reporting standards for quantitative research in psychology: The APA publications and Communications Board task force report. American Psychologist. 2018;73(1):3–25. 10.1037/amp0000191 29345484

[35] Toplak ME, West RF, Stanovich KE. Assessing miserly information processing: An expansion of the Cognitive Reflection Test. Thinking & Reasoning. 2014;20(2):147–68. 10.1080/13546783.2013.844729

[36] Thomson KS, Oppenheimer DM. Investigating an alternate form of the cognitive reflection test. Judgment and Decision Making. 2016;11(1):99–113.

[37] Pennycook G, Cheyne JA, Seli P, Koehler DJ, Fugelsang JA. Analytic cognitive style predicts religious and paranormal belief. Cognition. 2012;123(3):335–46. Epub 2012/04/07. 10.1016/j.cognition.2012.03.003 . 22481051

[38] De Neys W, Glumicic T. Conflict monitoring in dual process theories of thinking. Cognition. 2008;106(3):1248–99. Epub 2007/07/17. 10.1016/j.cognition.2007.06.002 . 17631876

[39] Bonner C, Newell BR. In conflict with ourselves? An investigation of heuristic and analytic processes in decision making. Memory & cognition. 2010;38(2):186–96. Epub 2010/02/23. 10.3758/mc.38.2.186 . 20173191

[40] Koehler DJ, James G. Probability matching and strategy availability. Memory & cognition. 2010;38(6):667–76. Epub 2010/09/21. 10.3758/mc.38.6.667 . 20852231

[41] Hart SG, Staveland LE. Development of NASA-TLX (Task Load Index): Results of Empirical and Theoretical Research In: Hancock PA, Meshkati N, editors. Advances in Psychology. 52: North-Holland; 1988 p. 139–83.

[42] Faul F, Erdfelder E, Lang AG, Buchner A. G*Power 3: a flexible statistical power analysis program for the social, behavioral, and biomedical sciences. Behavior research methods. 2007;39(2):175–91. Epub 2007/08/19. . 17695343 10.3758/bf03193146

[43] Golestani N, Alario FX, Meriaux S, Le Bihan D, Dehaene S, Pallier C. Syntax production in bilinguals. Neuropsychologia. 2006;44(7):1029–40. Epub 2006/01/24. 10.1016/j.neuropsychologia.2005.11.009 . 16427099

[44] Meyer A, Frederick S, Burnham TC, Guevara Pinto JD, Boyer TW, Ball LJ, et al Disfluent fonts don’t help people solve math problems. Journal of experimental psychology General. 2015;144(2):e16–30. Epub 2015/04/07. 10.1037/xge0000049 . 25844628

[45] Hilchey MD, Klein RM. Are there bilingual advantages on nonlinguistic interference tasks? Implications for the plasticity of executive control processes. Psychonomic bulletin & review. 2011;18(4):625–58. Epub 2011/06/16. 10.3758/s13423-011-0116-7 . 21674283

[46] de Zwaan L LC, Liu Y, Chardon T. Overconfidence in financial literacy: implications for planners. Financial Planning Research Journal 2017;3(2):31–46.

[47] Salthouse TA. The processing-speed theory of adult age differences in cognition. Psychological review. 1996;103(3):403–28. Epub 1996/07/01. 8759042 10.1037/0033-295x.103.3.403

[48] Lindenberger U, Mayr U, Kliegl R. Speed and intelligence in old age. Psychology and aging. 1993;8(2):207–20. Epub 1993/06/01. . 8323725 10.1037//0882-7974.8.2.207

[49] Li SC, Lindenberger U, Hommel B, Aschersleben G, Prinz W, Baltes PB. Transformations in the couplings among intellectual abilities and constituent cognitive processes across the life span. Psychological science. 2004;15(3):155–63. Epub 2004/03/17. 10.1111/j.0956-7976.2004.01503003.x . 15016286

[50] Bugg JM, Zook NA, DeLosh EL, Davalos DB, Davis HP. Age differences in fluid intelligence: contributions of general slowing and frontal decline. Brain and cognition. 2006;62(1):9–16. Epub 2006/04/11. 10.1016/j.bandc.2006.02.006 . 16603300

[51] Westbrook A, Kester D, Braver TS. What is the subjective cost of cognitive effort? Load, trait, and aging effects revealed by economic preference. PLoS One. 2013;8(7):e68210 Epub 2013/07/31. 10.1371/journal.pone.0068210 . 23894295 PMC3718823

[52] Stanovich KE, West RF. On the relative independence of thinking biases and cognitive ability. J Pers Soc Psychol. 2008;94(4):672–95. Epub 2008/03/26. 10.1037/0022-3514.94.4.672 . 18361678

[53] Zhang DC, Highhouse S, Rada TB. Explaining sex differences on the Cognitive Reflection Test. Personality and Individual Differences. 2016;101:425–7. 10.1016/j.paid.2016.06.034.

[54] Baron J, Scott S, Fincher K, Emlen Metz S. Why does the Cognitive Reflection Test (sometimes) predict utilitarian moral judgment (and other things)? Journal of Applied Research in Memory and Cognition. 2015;4(3):265–84. 10.1016/j.jarmac.2014.09.003.

[55] Bosch-Domènech A, Brañas-Garza P, Espín AM. Can exposure to prenatal sex hormones (2D:4D) predict cognitive reflection? Psychoneuroendocrinology. 2014;43:1–10. 10.1016/j.psyneuen.2014.01.023 24703165

[56] Capraro V. Does the truth come naturally? Time pressure increases honesty in one-shot deception games. Economics Letters. 2017;158:54–7. 10.1016/j.econlet.2017.06.015.

[57] Capraro V, Schulz J, Rand DG. Time Pressure and Honesty in a Deception Game. SSRN. 2018 10.2139/ssrn.3184537.

[58] Kool W, McGuire JT, Rosen ZB, Botvinick MM. Decision making and the avoidance of cognitive demand. Journal of experimental psychology General. 2010;139(4):665–82. Epub 2010/09/22. 10.1037/a0020198 . 20853993 PMC2970648

[59] Mækelæ MJ, Moritz S, Pfuhl G. Are Psychotic Experiences Related to Poorer Reflective Reasoning? Frontiers in Psychology. 2018;9:122 10.3389/fpsyg.2018.00122 29483886 PMC5816266

[60] Pfuhl G, Haghish EF, Biegler R. Assessment of altruism depends on inferred ulterior motives. Journal of Social, Evolutionary, and Cultural Psychology. 2013;7(1):36–50. 10.1037/h0099175. 2013-05135-005.

[61] Rand DG, Peysakhovich A, Kraft-Todd GT, Newman GE, Wurzbacher O, Nowak MA, et al Social heuristics shape intuitive cooperation. Nature Communications. 2014;5:3677 10.1038/ncomms4677 24751464

