# Correction: Deliberate reasoning is not affected by language

**DOI:** 10.1371/journal.pone.0213609

**Published:** 2019-03-05

**Authors:** Martin Jensen Mækelæ, Gerit Pfuhl

The first author's initials appear incorrectly in the citation. The correct citation is: Mækelæ MJ, Pfuhl G (2019) Deliberate reasoning is not affected by language. PLoS ONE 14(1): e0211428. https://doi.org/10.1371/journal.pone.0211428
